# Short- and long-term effects of essential oils on swine spermatozoa during liquid phase refrigeration

**DOI:** 10.1038/s41598-023-51030-2

**Published:** 2024-01-02

**Authors:** Ilaria Troisio, Martina Bertocchi, Domenico Ventrella, Maurizio Scozzoli, Maura Di Vito, Eleonora Truzzi, Stefania Benvenuti, Paola Mattarelli, Maria Laura Bacci, Alberto Elmi

**Affiliations:** 1 Department of Veterinary Medical Sciences, Alma Mater Studiorum - University of Bologna, Ozzano dell’Emilia, BO Italy; 2 Italian Society for Research on Essential Oils (Società Italiana per la Ricerca sugli Oli Essenziali—SIROE), Rome, RM Italy; 3 Department of Basic Biotechnological Sciences, Intensivological and Perioperative Clinics, Catholic University of the Sacred Heart, Rome, RM Italy; 4 Department of Chemical and Geological Sciences, University of Modena and Reggio Emilia, Modena, MO Italy; 5 Department of Life Sciences, University of Modena and Reggio Emilia, Modena, MO Italy; 6 Department of Agricultural and Food Sciences, Alma Mater Studiorum - University of Bologna, Bologna, BO Italy

**Keywords:** Animal physiology, Reproductive biology

## Abstract

The application of essential oils as potential alternatives to antibiotics in swine semen storage is promising, due to their antioxidant and antibacterial properties. However, detrimental effects on spermatozoa should be clarified first. The aim of this study was to evaluate 9 essential oils (EOs; *Satureja montana, Pelargonium graveolens, Cymbopogon nardus, Melaleuca leucadendron, Eucaliptus globulus, Citrus limon, Lavandula angustifolia, Lavandula hybrida, Mentha piperita*) and a blend (*GL mix*) on key morpho-functional parameters of swine spermatozoa. Test compounds were firstly chemo-characterized and experimental doses were prepared by suspending a fixed number of spermatozoa with 3 different concentrations (0.1, 0.5, 1 mg/mL) of EOs. Experimental doses were stored at 16 °C and sampled after 3 and 120 h for analysis. Overall, *S. montana*, *P. graveolens and L. angustifolia* EOs induced the strongest alterations, with *C. nardus* and *E. globulus* EOs being the best tolerated. Swine spermatozoa represent a good preliminary testing platform to screen toxicity and its different patterns. The comprehensive overview on the potential mechanisms of action of some of the most common EOs, despite of the direct aim of the study being swine reproduction, may be exploited in other fields of research within both veterinary and human medicine.

## Introduction

According to data reported by Lancet and released by WHO (World Health Organization), antimicrobial resistance (AMR) in bacteria caused an estimated 1.27 million deaths in 2019^1^, evidence that this is one of the most urgent matters in terms of public health. For this reason, the World health Assembly adopted a global action plan on antimicrobial resistance in 2015 and published a list of priority antibiotic-resistant bacteria in 2017 to identify the most relevant resistant bacteria at a global level for which there is an urgent need for new treatments^2^. The European Commission addressed the argument for the first time in 2001, with the first recommendation on the prudent use of antimicrobial agents in human medicine. Then, in 2011, proposed an actual action plane against the rising threats from antimicrobial resistance. In this circumstance, the Commission introduced the concept of One Health because of the indissoluble link between veterinary medicine, public health, and environmental sectors: *“In order to succeed a holistic approach is needed”*^3^. Finally, in the last year, the European Commission adopted a regulation to establish the criteria for the designation of antimicrobials to be reserved for the treatment of certain infections in humans^4^.

In animal husbandry, the use of antimicrobials has always been a common and widespread practice, even for preventive purposes, especially in intensive breeding. Lately, the restrictions imposed by the European Commission have led to the search for alternative preventative approaches such as vaccination, good biosafety practices, and high animal welfare conditions^5,6^. In some cases, finding alternatives has been complicated, as in the case of swine breeding and reproduction. In the swine industry, the extensive use of artificial insemination (AI) has contributed to the improvement of fertility performances, potentially one of the most important achievements in the livestock sector within the last 30 years^7^. This contributed to increasing in demand for seminal material from high pedigree boars so that today more than 93% of sows in pig producing countries are inseminated artificially^8^.

The most common bacterial populations found in boar semen are Gram-negative belonging to the Enterobacteriaceae family ^9–11^. In general, the incidence and relevance of certain bacteria species is correlated with seasonal conditions^12^ and specific sources of contamination: bacteria can come from both animal (*E. coli, Enterobacter spp., and Staphylococcus spp*.) and non-animal sources (*Pseudomonas, Bacillus,* and other species)^11^. Contamination points include boars, semen collection areas, water sources, thermometers, air handling systems and mainly poor personnel hygiene^9^.

Boar semen extenders are usually added with antibiotics to limit bacterial growth, capable of altering spermatozoa quality, since liquid preservation at 16 °C is still considered the best preservation technique^7,13^. This is related to the cold sensitivity of boar spermatozoa rich in unsaturated fat, and low tolerance to the most common cryopreservation additives^14^. Gentamicin, an aminoglycoside antibiotic, represents one of the most used preservative antimicrobials in porcine semen extenders as of today^12,15^, but gentamicin-resistant bacteria have been isolated since 2010 in European AI boar centers^16^. Recently, it has been emphasized that certain factors related to the age and the hygiene-sanitary of boars, as well as the methods used during semen collection, can significantly impact the presence of aerobic and coliform bacteria in semen^11^.

So that, a lot could be done with improvements in term of good practice of AI centers along the entire process from collection to the filling of AI doses^7^, as shown by an 8-years retrospective study conducted on 28 AI Centers in Europe after the identification and introduction of 9 HCCPs (hygienic critical control points) and the evaluation of hygienic conditions. The analyses show that hygiene management has contributed to reduce contamination of extended ejaculates; in particular, the bacterial contamination rate decreased by 13.5% between audit period 1 and 4^15^. It is important to clarify that most of bacteria detected are considered nonpathogenic, nevertheless high levels of bacterial contamination can lead to negative consequences^9^. High bacterial counts (over 1.4 × 10^4^ CFU/mL) lead to decreased sperm motility and changes in pH. In some cases, bacteria can affect the integrity of sperm membrane which results in reduced viability and damaged acrosomes. Additionally, bacterial concentration and storage time can influence the mitochondrial membrane potential of sperm. Lastly, it was demonstrated that some bacteria can cause agglutination of cells, interfering with motility^12^. Several studies in recent years have focused on the need for finding new strategies to avoid using antibiotics in the extenders. Out of physical methods, such as ultracentrifugation, and microfiltration of seminal plasma^17^ that have shown excellent results but turned out to be really expensive. There are also studies that show that colloid centrifugation can reduce bacterial contamination of boar semen without the use of antimicrobials^18^, and recently there are some evidences that single-layer centrifugation could enhance chromatin structure in boar semen^19^. Another approach is represented by antimicrobial peptides^20^: substances that can alter the bacterial membrane, potentially killing pathogens.

Among these different alternatives, there are also Essential Oils (EOs): products of the secondary metabolism of plants, which are aromatic and volatile substances responsible of the characteristic fragrance of the plant. Essential oils consist of a mixture of different terpenes, sesquiterpenes and aromatic compounds such as phenols and phenylpropanes^21^. By using different extraction methods (distillation, mechanical pressure, extraction using solvent)^22,23^ is it possible to obtain EOs in different compositions. A lot of them have proven antifungal, antiviral, and also antibacterial potentials, as reported by Tariq and colleagues^24^, with an exhaustive review in which are listed the mechanisms of action of the most famous compound among the most widely studied.

The aim of the present study was to evaluate 9 EOs (*Satureja montana, Pelargonium graveolens, Cymbopogon nardus, Melaleuca leucadendron, Eucaliptus globulus, Citrus limon, Lavandula angustifolia, Lavandula hybrida, Mentha piperita*) and a blend (*GL mix*) for short and long term potential toxic effects on porcine spermatozoa by evaluating main morpho-functional parameters (viability, acrosomal reactions and total motility). The activity of essential oils on porcine spermatozoa has also been assessed with previous studies in which it has been already tested the potential use of some officinalis Essential Oils as antimicrobial agents for liquid storage^13,25,26^.

The outcome of the sequent study may deepen the knowledge around these compounds that can become interesting new proposals as new alternatives for the liquid short and long storage of seminal porcine doses contributing towards fight against antimicrobial resistance.

## Results

The results of the chemo-characterization of the different EOs used in the present study are reported in the Supplementary Materials (Tables S1–S10).

The effects of both short- (3 h) and long-term (120 h) exposure to the different EOs on porcine spermatozoa morpho-functional parameters are represented, including the results of the Dunnett’s tests used to compare EO-treated samples to the control in the following figures**.** An initial graphical overview of the effects of all test compounds is reported in the Supplementary Materials (Figure S1). However, to better describe the results, the different EOs were divided into subgroups according to the different patterns of toxicity exhibited.

### *Satureja montana*, *Pelargonium graveolens* and *Lavandula angustifolia*

**Figure 1:**
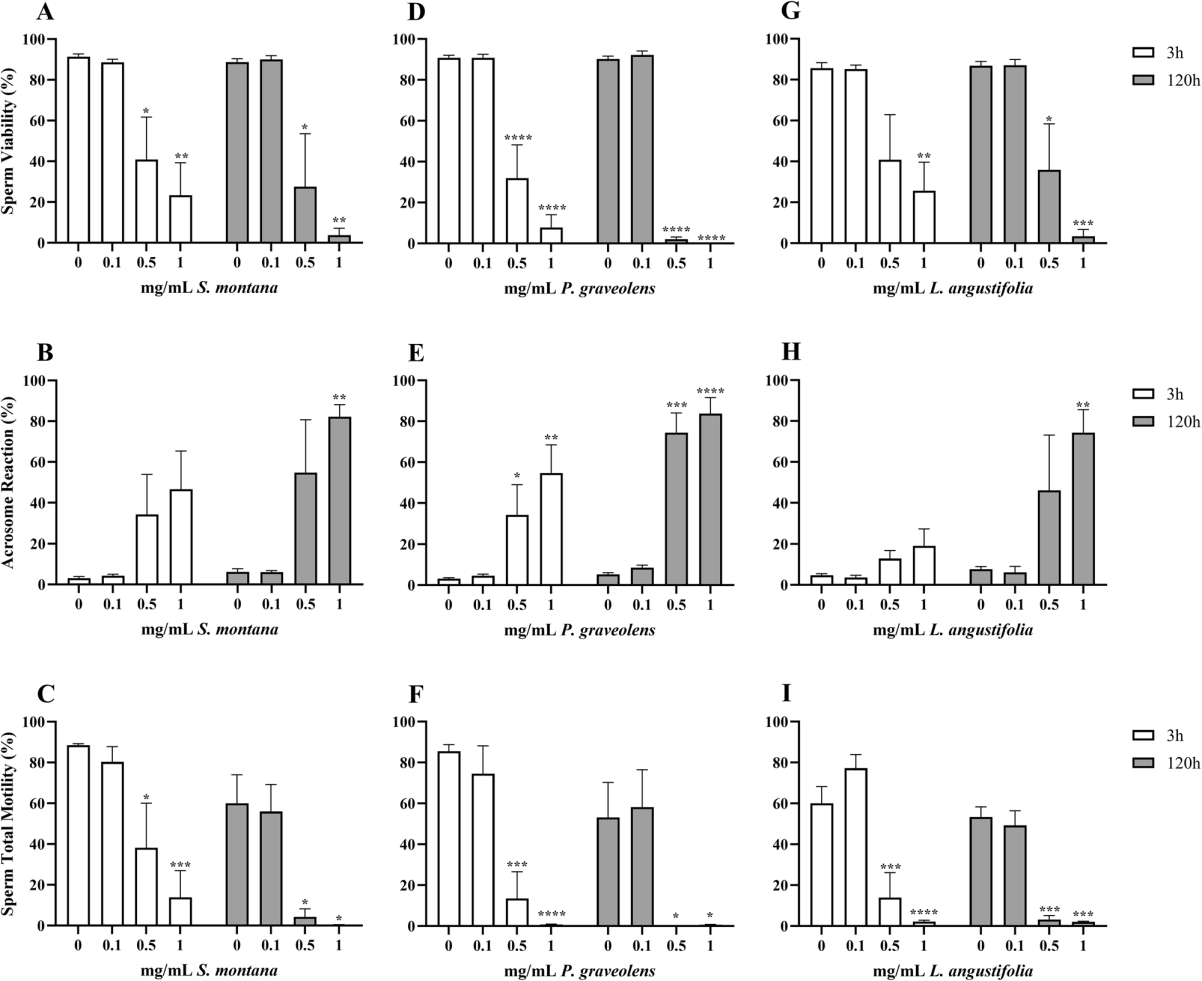
Effects of *S. montana* (**A**–**C**), *P. graveolens* (**D**–**F**) and *L. angustifolia* (**G**–**I**) EOs on sperm viability (**A**,** D**,** G**), acrosome reaction (**B**,** E**,** H**) and sperm total motility (**C**,** F**,** I**). Data are expressed as mean ± standard error of the mean. 0 = control samples (only emulsifiers). * = *p* < 0.05; ** = *p* < 0.01; *** = *p* < 0.001; **** = *p* < 0.0001.

These EOs statistically altered all morpho-functional parameters, as shown in Fig. 1.

The 2 way-ANOVA showed how treatment with *S. montana* altered Viability (V; *p* = 0.0001), Acrosome Reaction (AR; *p* = 0.0005), and Sperm Total Motility (TotM; *p* < 0.0001), while storage time only TotM (*p* = 0.0107). Upon comparison with the control, V (Fig. 1A) was statistically impaired by the middle and the highest concentrations at both timepoints: 0.5 mg/mL (3 h: *p* = 0.0257, 120 h: *p* = 0.0190), 1 mg/mL (3 h: *p* = 0.0028; 120 h: *p* = 0.0013). The same applies to TotM (Fig. 1C): 0.5 mg/mL (3 h: *p* = 0.0166; 120 h: *p* = 0.0221), 1 mg/mL (3 h: *p* = 0.0006; 120 h: *p* = 0.141). On the other hand, AR (Fig. 1B) was only worsened by 1 mg/mL of EO at 120 h (*p* = 0.0046).

Treatment with *P. graveolens* EO statistically altered all three parameters (V, AR and TotM: *p* < 0.0001), while storage time only influenced AR (*p* = 0.0093). The post hoc analysis showed that V (Fig. 1D) was significantly impaired upon exposure to 0.5 and 1 mg/mL of EO at both timepoints (*p* < 0.0001), just like AR (0.5 mg/mL: 3 h *p* = 0.0467, 120 h *p* = 0.0002; 1 mg/mL: 3 h *p* = 0.0011, 120 h *p* < 0.0001; Fig. 1E) and TotM (0.5 mg/mL: 3 h *p* = 0.0002, 120 h *p* = 0.0116; 1 mg/mL: 3 h *p* = 0.0002, 120 h *p* = 0.0116; Fig. 1F).

At last, while treatment with *L. angustifolia* EO influenced all parameters (V and TotM: *p* < 0.0001; AR: *p* = 0.0037), storage time only affected TotM and AR (respectively: *p* = 0.0078 and *p* = 0.0247). The results of Dunnett’s tests, in this case, showed that V (Fig. 1G) was statistically reduced at 3 h only by 1 mg/mL (*p* = 0.0092), while by both 0.5 (*p* = 0.0265) and 1 mg/mL (*p* = 0.0006) at 120 h. TotM (Fig. 1I) was compromised at both timepoints upon exposure to 0.5 (3 h: *p* = 0.0004; 120 h: *p* = 0.0002) and 1 mg/mL (3 and 120 h: *p* = 0.0001). On the other hand, AR (Fig. 1H) was only impaired by the highest concentration, after 120 h of exposure impaired (*p* = 0.0015).

### *Lavandula hybrida* and *Citrus limon*

**Figure 2:**
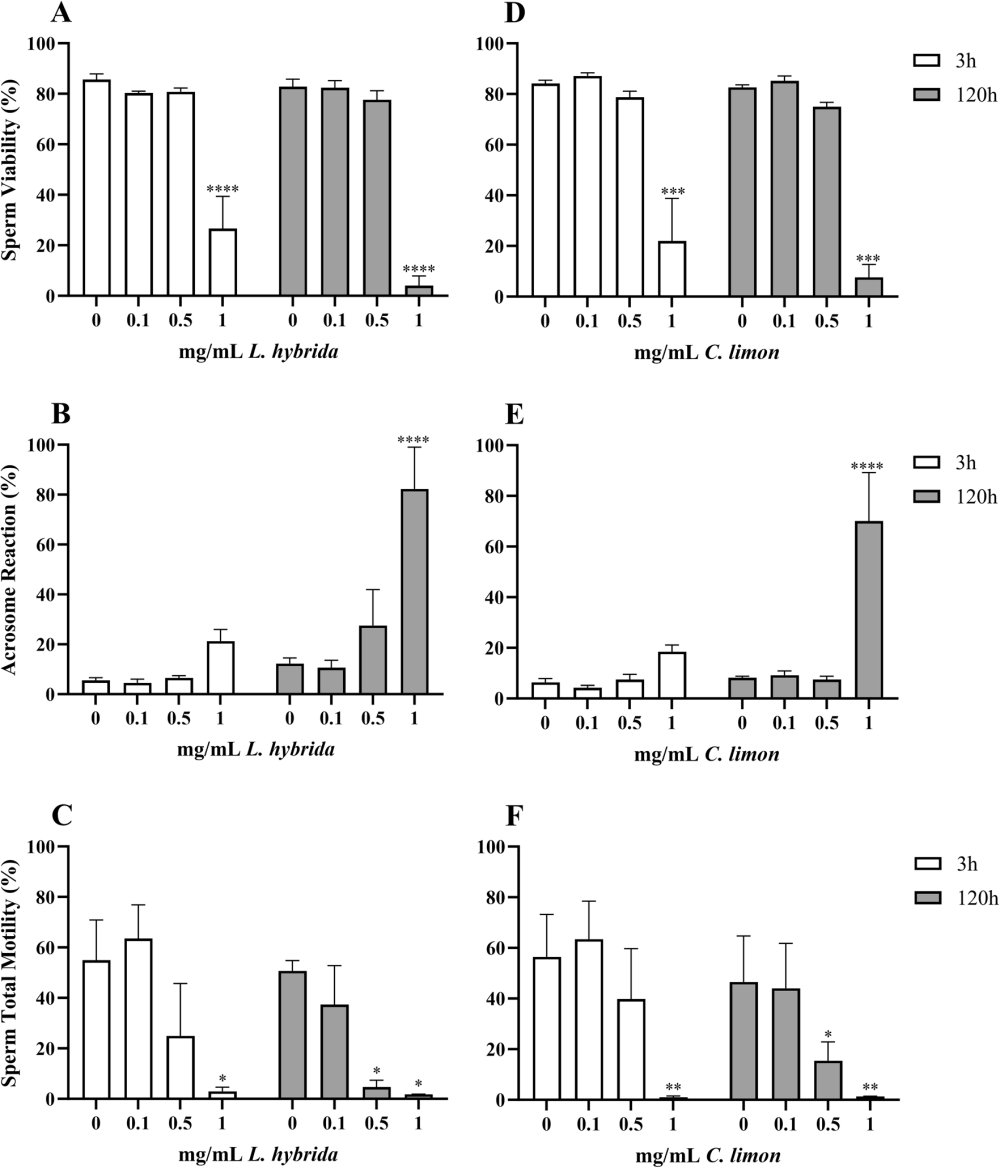
Effects of *L. hybrida* (**A–C**) and *C. limon* (**D–F**) EOs on sperm viability (**A**,** D**), acrosome reaction (**B**,** E**) and sperm total motility (**C**,** F**). Data are expressed as mean ± standard error of the mean. 0 = control samples (only emulsifiers). * = *p* < 0.05; ** = *p* < 0.01; *** = *p* < 0.001; **** = *p* < 0.0001.

The EOs obtained from *L. hybrida* and *C. limon* showed a very similar pattern of actions on spermatozoa, represented in Fig. 2.

The 2 way-ANOVA showed that treatment with both EOs reduced V (*p* < 0.0001) (Fig. 2A,D) and increased AR (*p* = 0.0001) (Fig. 2B,E). Acrosomal reactions, in both cases, were statistically increased also by storage time (*L. hybrida* EO: *p* = 0.0008; *C. limon* EO: *p* = 0.0092) and interaction between variables (*L. hybrida* EO: *p* = 0.0121; *C. limon* EO: *p* = 0.0051). Also TotM (Fig. 2C,F) was compromised by the treatments (*L. hybrida* EO: *p* < 0.0009; *C. limon* EO: *p* = 0.0002). The post hoc analysis showed that V was statistically altered at both timepoints upon exposure to 1 mg/mL (*p* = 0.0001), while AR was only worsened at 120 h for both EOs (*p* < 0.0001). Upon comparison with the controls, TotM showed a slightly different trend: 0.5 mg/mL (120 h: *L. hybrida* EO *p* = 0.0368, *C. limon* EO *p* = 0.0303), 1 mg/mL (3 h: *L. hybrida* EO *p* = 0.0181, *C. limon* EO *p* = 0.0015; 120 h: *L. hybrida* EO *p* = 0.0259, *C. limon* EO *p* = 0.0032).

### *Mentha piperita*, *Melaleuca leucadendron* and *GL mix*

**Figure 3:**
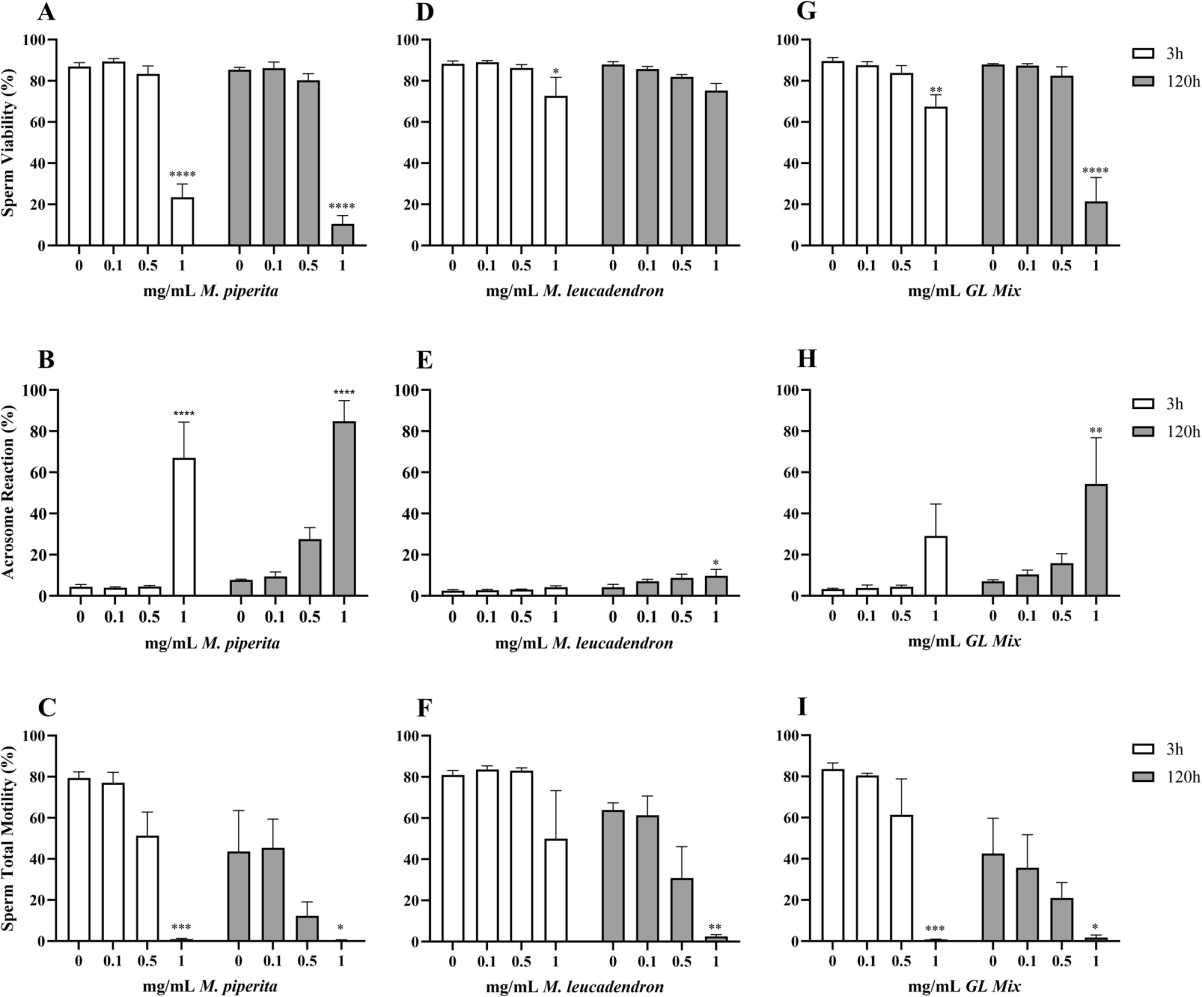
Effects of *M. piperita* (**A**–**C**) and *M. Leucadendron* (**D**–**F**) EOs and *GL Mix* (**G-I**) on sperm viability (**A**,** D**,** G**), acrosome reaction (**B**,** E**,** H**) and sperm total motility (**C**,** F**,** I**). Data are expressed as mean ± standard error of the mean. 0 = control samples (only emulsifiers). * = *p* < 0.05; ** = *p* < 0.01; *** = *p* < 0.001; **** = *p* < 0.0001.

These EOs and the blend only induced statistically relevant alterations when used at the highest concentration of 1 mg/mL (Fig. 3).

The results of the 2way-ANOVA for the *M. piperita* EO showed that all three parameters were statistically affected by the treatment (*p* = 0.0001), but only AR and TotM by storage time (respectively: *p* = 0.0031 and *p* = 0.0017). The Dunnett’s tests highlighted how, for both timepoints, only the samples treated with the highest concentration of EO statistically differed from the control samples: at 3 h *p* < 0.0001 for all three parameters, at 120 h *p* < 0.0001 for V (Fig. 3A) and AR (Fig. 3B), and *p* = 0.0202 for TotM (Fig. 3C).

Treatment with *M. leucadendron* EO impaired V (*p* = 0.0042; Fig. 3D) and TotM (*p* = 0.0013; Fig. 3F), while storage time altered AR (*p* = 0.0009; Fig. 3E) and TotM (*p* = 0.0003). The post hoc analysis showed that V was significantly worsened by 1 mg/mL at the first timepoint (*p* = 0.0206). For the other two parameters, alterations were only recorded after 120 h, still with the higher concentration (AR: *p* = 0.0492, TotM: *p* = 0.0022).

The 2way-ANOVA showed that treatment with *GL mix* statistically altered: V (*p* < 0.0001; Fig. 3G), AR (*p* = 0.0056; Fig. 3H) and TotM (*p* = 0.0001; Fig. 3I). Viability was also influenced by storage time (*p* = 0.0006) and by the interaction between the variables (*p* = 0.0001); storage time was statistically relevant also on TotM (*p* = 0.0008). Dunnett’s tests highlighted that samples added with 1 mg/mL of *GL mix* showed different alterations when compared to the control: at 3 h for V (*p* = 0.0034) and TotM (*p* = 0.0002), at 120 h for all parameters (V: *p* < 0.0001, TotM: *p* = 0.0424, AR: *p* = 0.0098).

### *Cymbopogon nardus* and *Eucaliptus globulus*

**Figure 4:**
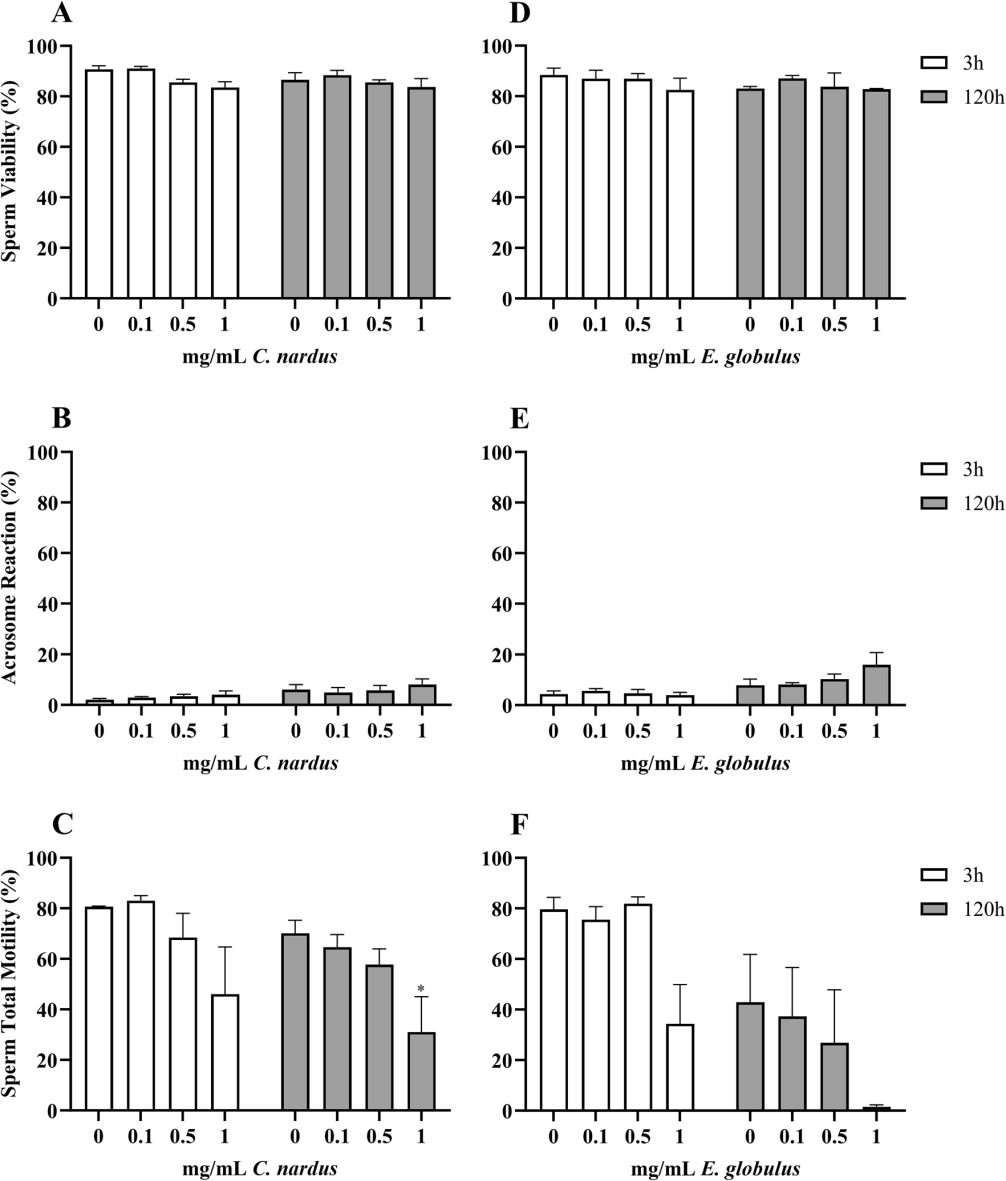
Effects of *C. nardus* (**A**–**C**) and *E. globulus* (**D**–**F**) EOs on sperm viability (**A**), acrosome reaction (**B**) and sperm total motility (**C**). Data are expressed as mean ± standard error of the mean. 0 = control samples (only emulsifiers). * = *p* < 0.05; ** = *p* < 0.01; *** = *p* < 0.001; **** = *p* < 0.0001.

These EOs showed minimal to no toxic effects on swine spermatozoa both during short- and long-term storage (Fig. 4).

As for *C. nardus* EO, the 2way analysis of variance showed that there were no interferences on V (Fig. 4A), while storage time statistically impaired AR (*p* = 0.0142; Fig. 4B) and TotM (*p* = 0.0049; Fig. 4C). Upon comparison with the control, the latter was only statistically worsened by 1 mg/mL of EO at 120 h (*p* = 0.0277).

Similarly, looking at the results for the EO of *E. globulus*, V was never impaired (Fig. 4D), while AR (Fig. 4E) and TotM (Fig. 4F) only by storage time (respectively *p* = 0.0019 and *p* = 0.0002).

## Discussion

The analyses conducted on boar spermatozoa do provide not only necessary preliminary information regarding the potential use of EOs in swine AI seminal doses, but also supply general insights into the different mechanisms of action underlying their biological properties. In particular, the chosen parameters provide data on two key damage mechanisms: membrane disruption (as indicated by viability and percentage of reacted acrosomes) and mitochondrial activity impairment by means of membrane depolarization, potentially leading to the loss of motility^27^. All of the above support the hypothesis that swine spermatozoa, due to their ease of collection and low ethical value, may represent a useful preliminary screening platform for natural substances toxicity. The reason for choosing to evaluate the effects of EOs at two different timepoints (3 and 120 h) is to simulate the usual short- and long-term storage conditions of swine ejaculates at the standard temperature of 16 ± 1 °C. The assessments conducted after 3 h of incubation should provide information regarding the immediate, direct effects of EOs on spermatozoa, while the ones after 120 h should also provide insights regarding the lasting capabilities of the EOs themselves and their interaction with the given environment. As for the tested concentrations, the 3 chosen one should represent a high one, potentially clearly showing detrimental effects, a low one, still regarded as potentially active against contaminants according to literature but as low as possible, and a middle one.

Overall, it is possible to identify a trend of concentration related effects, that is in according to what was already reported for previous studies EOs such as *R. officinalis* and *T. capitata*^26^, and *M. Alternifolia*^13^. However, no evidence of damage was highlighted for all the tested compounds when using the lowest concentration of 0.1 mg/mL, both looking at membranes’ alterations and motility. This consideration is important because the literature shows different works proving that some of the EOs analysed in this study already have antibacterial and antifungal effects at very low concentrations, below those considered able to alter morpho-functional sperm parameters.

It can be stated that the first group of EOs (*S. montana*, *P. graveolens* and *L. angustifolia)* exhibits similarities in very strong impairment of sperm quality starting from the concentrations of 0.5 mg/mL. Additionally, it seems that the storage period does not significantly influence viability, whereas in other cases, it appears to have significant effects. The EO obtained from *S. montana* has proven antibacterial activity at a concentration lower than that considered toxic for boar spermatozoa. In particular, as reported by literature, *S. montana* EO reports a MIC (minimum inhibitory concentration) value of 0.39 mg/mL towards *Staphylococcus aureus*^28^, where MIC is the lowest concentration required by antimicrobials to clearly inhibit the growth of a bacterium after overnight incubation^29^, and MICs far < 0.5 mg/mL also against *Pseudomonas aeruginosa, Streptococcus pyogenes, Streptococcus mutans, Streptococcus sanguis, Streptococcus salivarius, Enterococcus faecalis, Lactobacillus acidophilus*^30^. All of bacteria mentioned above have been isolated from neat boar ejaculates^9^. Therefore, out of the tested EOs, *S. montana* would be a very good candidate for continuing investigating its potential use for swine AI doses.

The second group consisted of *L. hybrida* and *C. limon* EOs. *Lavandula hybrida* is also known as *Lavandula x intermedia*, and is a sterile hybrid of true lavender (*L. angustifolia*) and spike lavender (*L. latifolia*)^31^. Comparative studies between *L. x intermedia* and *L. angustifolia* are available: the former possesses similar or stronger antibacterial and antifungal effects than true lavender oil, in particular against Candida spp. The analysis of antimicrobial activity against oral pathogenic bacteria showed that lavandin oil has MIC values ranging from 0.002 to 0.512 mg/mL^32^. Moreover, *L. hybrida* yields more EOs per kg than *L. Angustifolia*, making it also a cheaper alternative to the true lavender oil^31^. *L. hybrida* and *C. limon* EOs were shown together because of the evident common pattern of alteration. Specifically, viability was only influenced by the highest concentration tested (1 mg/mL), without being affected by storage time. On the other hand, the number of reacted acrosomes significantly increased not only due to 1 mg/mL of both EOs, but the storage period played a decisive role in triggering the capacitation process. Different considerations should be made for motility since, in the case of *L. hybrida*, time did not seem to have any effect, while did for *C. Limon* EO. Overall, this was the only parameter statistically influenced already at the middle concentration (0.5 mg/mL) in this second group, although only after 120 h in both cases. When comparing these findings with the available literature, it shows that *L. hybrida* EO has antifungal activity at very low concentrations and that *C. limon* EO can successfully inhibit the development of *L. monocytogenes* in minced beef meat already at 0.06–0.312 mg/g^33^*.*

The EOs reported in the third group (*M. piperita, M. leucadendron* and *GL mix*) only show morpho-functional impairment upon treatment with the highest concentration of 1 mg/mL. In particular, for *M. piperita* EO, the concentrations of 0.1 and 0.5 mg/mL seem to be very well tolerated, with time storage being relevant only for acrosomal reactions and total motility. This EO has been tested against *S. aureus*, *S. pyogenes* and *S. mutans*, with outcoming MICs of approximately 0.6 mg/mL^34^. Such values are promising, since they are close to the well tolerated concentration of 0.5 mg/mL, but further studies increasing the tested concentration > 0.5 < 1 mg/mL would allow for more accurate applications. Again, *Melaleuca Leucadendron* EO and *GL mix* were only capable of altering spermatozoa upon treatment with 1 mg/mL, but with less intense damages when assessed against *M. piperita.* Not a lot of literature is available for *M. leucadendron*, while *GL mix* is a patent-pending mixture of different EOs, therefore to better understand their potential, further studies are needed.

Proceeding towards the last two tested EOs, it is clear that the pattern of toxicity becomes less and less relevant. *C. nardus* EO seems to be very well tolerated at all tested concentrations, despite a mild reduction in total motility, but only after 120 h of incubation. The hypothesis, in this case, is that such effect at 1 mg/mL may be mediated by a mild interaction with mitochondrial function leading to disruption of cellular energy metabolism thus reduced energy production and cellular dysfunction. As for *E. globulus* EO, the post-hoc tests have highlighted no differences between treated samples and control ones. In this case, the increase of acrosomal reactions and the loss of motility detected is only accounted by the storage time. It still has to be acknowledged that the lack of statistical significance for motility at 120 h upon treatment with 1 mg/mL, potentially due to the statistical approach used and the sample size, does not imply a lack of biological relevance since, indeed, motility is almost completely suppressed. Nonetheless, literature shows a good amount of work that proves antibacterial activity of this EO, both alone and in combination with other agents, even against strains of *methicillin-resistant Staphylococcus aureus* (MRSA) bacteria, with MIC values ranging from 0.032 to 10 mg/mL^35^.

To discuss the overall results of the present work, it is important to state that the exact composition of the different EOs changes not only from plant to plant, but also within the same plant during the different phases of its growth cycle. This is why, when investigating and reporting data for EOs, assessing and taking into account their exact composition is pivotal. The complete chemo-characterization of the test compounds is reported in the Supplementary Material (Tables S1–S10). As reported by the tables, our EOs are rich in terpenes (also known as terpenoids or isoprenoids), the largest group of natural compounds, with approximately 25,000 structures reported^36^. Terpenes can have a variety of biological activities, such as antibacterial, antifungal, anti-inflammatory, antioxidant properties. These potential properties of terpenes are currently the subject of numerous scientific studies^37–40^, but only a limited amount of these consider the possible use in preserving the quality of semen during storage, thanks to their antimicrobial and antioxidant properties. Nonetheless, a recent work has shone a light on the role of carvacrol as a mitigating agent for the reduction of boar semen quality during storage under cooling conditions: its ability to decrease the production of oxygen reactive species and regulate mitochondrial activity in porcine sperm makes it a promising antioxidant^41^. Carvacrol is a terpene belonging to the class of monoterpenes, formed by coupling two units of isoprene (C10) and is a component found in various plants. As all terpenes, carvacrol also shows concentration-dependent activity, with higher concentrations leading to harmful effects. Amongst the EOs used in this work, *S. montana* showed the highest carvacrol content (52.56%, Table S1), and was indeed one of the test compounds that induced the strongest alterations on morpho-functional parameters, supporting the predominant biological activity of carvacrol itself. Upon literature search, it seems understandable why this particular EO also shows the lowest MICs for different bacterial populations. However, it is important to note that EOs are complex mixtures made up of many molecules, potentially with both synergic and antagonist effects between each other, thus their proprieties strongly depend on their combination with other compounds, as reported by Bakkali and colleagues^42^. This peculiarity was demonstrated by Elmi and colleagues^13^, with a comparative study between tea tree oil (*Melaleuca alternifolia* EO) and its principal component, terpinen-4-ol, on the morpho-functional parameters of swine spermatozoa. The results showed how, despite terpinen-4-ol accounted for > 40% of the used *M. alternifolia* EO, the toxicity patterns were very different and to be ascribable to some synergistic interaction between other constituent compounds. In view of above, since the concept of synergy seems to be extremely relevant, it is not possible to translate results obtained by the use of isolated constituents to the whole mixture within the given EO. Unfortunately, despite all the good potential capabilities, such differences and the need for specific studies and tests on each single batch of EO, represent the main pitfall of their applications.

As for the comparison of the MICs of the different tested EOs against the most common bacteria found in porcine ejaculates, the discussion can be challenging as, according to different studies, the populations of contaminating bacteria are extremely different and various. In addition, each and every batch of EOs will provide different results. Dedicated studies would help shining a light on this matter yet, based on the results hereby presented, it looks like the EO derived from *S. montana* is the most characterized in terms of specific antibacterial capabilities and is active against the majority of the most common bacterial population found in boar ejaculates.

In conclusion, the results of the present study provide a comprehensive overview of the potential mechanisms of action of some of the most common EOs, especially concerning their interaction with porcine male gametes. The most promising ones will now have to undergo testing, as already done for other EOs such as *T. capitata* and *R. officinalis*^25^, for their antimicrobial capabilities directly in swine artificial insemination doses. Regardless of the direct aim of the study being swine reproduction, results may be exploited in other fields of research within both veterinary and human medicine.

## Materials and methods

### Natural substances and reagents

Nine pure EOs and one undisclosed blend provided by APA-CT (Forlì, Italy) were used for the present study, in particular: *Satureja montana, Pelargonium graveolens, Cymbopogon nardus, Melaleuca leucadendron, Eucaliptus globulus, Citrus limon, Lavandula angustifolia, Lavandula hybrida* and *Mentha piperita* EOs*,* and *GL mix*. The *GL mix* is a patent-pending solution of nine EOs: *Eucaliptus globulus, Satureja hortensis, Citrus aurantium var. dulcis, Thymus vulgaris, Melaleuca alternifolia, Citrus limon, Lavandula hybrida, Melaleuca leucadendron and Thymus capitatus*, dispersed in Glyceryl polyethyleneglycol ricinoleate^27^. The natural substances were kept at 4 °C, in darkened glass bottles to avoid alterations. One aliquot of each substance was used for the chemo-characterization, while, for the in vitro experiments, they were added with 0.5% dimethylglyoxime (DMSO) and Tween 80 (0.002%) to grant uniform emulsification^43^.

### Chemo-characterization of EOs

The chemo-characterization of EOs was performed according to previously published protocols^25^, upon Gas Chromatography-Mass Detector (GC–MS) analysis and Gas Chromatography-Flame Ionization Detector (GC-FID). Qualitative analysis was performed using an Agilent Technologies HP-5 MS cross-linked poly-5% diphenyl–95% dimethyl polysiloxane (30 m × 0.25 mm i.d., 0.25 μm film thickness) capillary column on a 7890A gas chromatograph coupled with a 5975C network mass spectrometer. The semi-quantitative characterization of EOs was carried out on a HP-5 cross-linked poly-5% diphenyl–95% dimethyl polysiloxane (30 m × 0.32 mm i.d., 0.25 μm film thickness) capillary column on a 7890A gas chromatograph with a flame ionization detector (Agilent Technologies, Waldbronn, DE). Compounds were identified by comparing the retention times of the chromatographic peaks with those of authentic reference standards run under the same conditions, the fragmentation spectra, and the linear retention indices (LRIs) relative to C_8_–C_40_ n-alkanes obtained on the HP-5 column under the above-mentioned conditions with the literature reference^44^.

### Porcine spermatozoa collection and evaluations

To estimate cytotoxicity on porcine spermatozoa, the EOs, and *GL mix* were evaluated as formerly reported in other works^13,27^.

Three adult boars (Large White × Duroc), 1–2 years old, with a weight ranging from 220 to 250 kg were enrolled for this experimental protocol as ejaculate donors, housed in single pens as dictated by the National law (D.lgs n.122/2011). Semen was collected twice a week by an experienced operator using the hand-gloved technique in a pre-heated (37 °C) thermos, as previously described ^13^.

Semen collection is considered as a zootechnical routine practice, and does not classify as procedure according to the Lgs. Decree 26/2014. Therefore, no ethical approval was needed for the present study.

After the collection, the sperm-rich fraction (SFR) of each ejaculate was immediately diluted 1:1 (v/v) with an in-house prepared swine fertilization medium (SFM) without any antibiotic^13,45^. The experimental doses were prepared by suspending a fixed number of spermatozoa (15 × 10^7^ spz) in 5 mL of SFM (final concentration = 3 × 10^7^ spz/mL) with 3 different concentrations (1, 0.5 and 0.1 mg/mL) of EOs previously added with emulsifiers as described above. For each experiment, control samples were realized by only adding the emulsifiers. After preparation, the experimental doses were stored for 120 h in a refrigerated bath (AD28R-30, VWR International S.r.l., Milano, IT), set at 16 °C (± 1 °C) and sampled at 3 and 120 h^26,27^.

Each test compound was tested on three different ejaculates by each boar for key morpho-functional parameters following previously published protocols.

Viability (V) was assessed by eosin-nigrosin staining. Briefly, 10 µL of the staining solution were mixed with 10 µL of each dose, and 8 µL were immediately smeared on a glass slide. The percentage of live cells (undyed spermatozoa/all spermatozoa) was calculated on a minimum of 200 cells^26^, upon microscopy evaluation (Eclipse E600, 40×, Nikon, Tokyo, JP).

The percentage of reacted acrosomes (AR) was assessed using modified Coomassie Blue staining: spermatozoa were fixed using 4% formaldehyde, washed, and suspended in ammonium acetate before being smeared onto a microscope slide and incubated with Coomassie Blue G250 staining solution (0.22%). The percentage of reacted acrosomes (undyed acrosomes/all acrosomes) was calculated from a minimum of 200 cells, upon microscopy evaluation^26^. All slides (both V and AR) were coded and analyzed by a blinded operator to avoid biases.

Total motility (TotM) was analysed by computer-assisted sperm analysis (CASA; Hamilton Thorne CEROS II; Animal Motility II, Software Version 1.9, Beverly, MA, USA), using heated dedicated slides (Leja 4 chamber slides, Leja, IMV technologies, L’Aigle, FR).

As inclusion criteria, only SRFs with V > 85% and TotM > 80% were used for the experimental protocol^27^.

### Statistical analysis

The statistical analyses were performed using the software GraphPad Prism v.8 (GraphPad Software Inc., San Diego, CA, USA). In order to analyze the results of each parameter and each test compound, 2way-ANOVAs were performed setting treatment, time storage and their interaction as factors. Post-hoc analyses were performed by means of Dunnett’s tests, to assess differences between the control samples and the ones treated with different concentrations of test compounds. Significance was set at *p* < 0.05.

### Supplementary Information

Supplementary Information.

## Data Availability

The data of this manuscript are available from the corresponding author upon reasonable request.
