# Supplementary material for: Short- and long-term effects of essential oils on swine spermatozoa during liquid phase refrigeration
Source: Sci Rep. 2024 Jan 2;14:285. doi: 10.1038/s41598-023-51030-2 (PMC10762118; doi:10.1038/s41598-023-51030-2)
Supplement: Supplementary file 1 — Supplementary Information. [file 41598_2023_51030_MOESM1_ESM.docx]

# SUPPLEMENTARY MATERIAL

**Short- and long-term effects of Essential Oils on swine spermatozoa during liquid phase refrigeration**

Ilaria Troisio, Martina Bertocchi, Domenico Ventrella, Maurizio Scozzoli, Maura Di Vito, Eleonora Truzzi, Stefania Benvenuti, Paola Mattarelli, Maria Laura Bacci, Alberto Elmi.

**Table S1.** Composition of the essential oil of *Satureja montana*.

| **Compound** | **Lit. LRI** | **Exp. LRI** | **Area %** |
| --- | --- | --- | --- |
| carvacrol | 1301 | 1305 | 52.56 |
| *p*-cymene | 1024 | 1029 | 13.79 |
| thymol | 1295 | 1298 | 6.66 |
| γ-terpinene | 1060 | 1061 | 5.33 |
| neral | 1245 | 1252 | 3.85 |
| β-caryophyllene | 1420 | 1424 | 3.11 |
| borneol | 1066 | 1168 | 2.19 |
| myrcene | 989 | 993 | 1.24 |
| α-pinene | 936 | 934 | 1.15 |
| linalool | 1099 | 1102 | 0.8 |
| α-terpinene | 1017 | 1017 | 0.75 |
| octen-3-ol | 981 | 980 | 0.74 |
| α-thujene | 928 | 927 | 0.71 |
| limonene | 1029 | 1030 | 0.68 |
| camphene | 950 | 948 | 0.65 |
| β-bisabolone | 1508 | 1513 | 0.65 |
| terpinen-4-ol | 1177 | 1179 | 0.49 |
| camphor | 1143 | 1147 | 0.41 |
| geranial | 1278 | 1275 | 0.29 |
| *cis*-β-ocimene | 1038 | 1039 | 0.24 |
| caryophyllene oxide | 1589 | 1594 | 0.24 |
| *trans*-β-ocimene | 1051 | 1049 | 0.22 |
| *trans*-sabinene hydrate | 1064 | 1067 | 0.2 |
| α-terpineol | 1190 | 1192 | 0.16 |
| terpinolene | 1090 | 1089 | 0.15 |
| α-phellandrene | 1004 | 1005 | 0.14 |
| β-pinene | 975 | 976 | 0.11 |
| δ-cadinene | 1524 | 1521 | 0.1 |
| α-muurolene | 1499 | 1502 | 0.07 |
| γ-muurolene | 1477 | 1484 | 0.04 |
| γ-cadinene | 1523 | 1522 | 0.04 |
| sabinene | 973 | 974 | 0.01 |
| **TOTAL** | | | **97.80** |

Experimental retention indices and literature retention indices (HP-5 column) according to NIST 14 (National Institute of Standards and Technology, USA; 14^th^ edition) library database [1], Babushok et al. [2].

**Table S2.** Composition of the essential oil of *Pelargonium graveolens*.

| **Compound** | | **Lit. LRI** | **Exp. LRI** | **Area %** |
| --- | --- | --- | --- | --- |
| citronellol | | 1237 | 1237 | 33.77 |
| geraniol | | 1259 | 1265 | 33.38 |
| geranial | | 1278 | 1283 | 3.74 |
| geranyl formate | | 1300 | 1307 | 3.17 |
| isomenthone | | 1164 | 1166 | 2.79 |
| linalool | | 1099 | 1102 | 2.69 |
| γ-eudesmol | | 1630 | 1633 | 2.1 |
| menthone | | 1154 | 1155 | 1.67 |
| δ-cadinene | | 1524 | 1520 | 1.21 |
| *cis*-rose oxide | | 1112 | 1112 | 0.84 |
| β-caryophyllene | | 1420 | 1425 | 0.79 |
| phenylethyl tiglate | | 1587 | 1591 | 0.68 |
| β-bourbonene | | 1384 | 1389 | 0.67 |
| geranyl butanoate | | 1562 | 1563 | 0.57 |
| germacrene D | | 1481 | 1486 | 0.55 |
| viridiflorene | | 1496 | 1501 | 0.41 |
| *trans*-rose oxide | | 1128 | 1128 | 0.34 |
| α-copaene | | 1376 | 1380 | 0.33 |
| α-pinene | | 936 | 932 | 0.32 |
| aromadendrene | | 1440 | 1446 | 0.31 |
| neral | | 1245 | 1249 | 0.28 |
| α-cubebene | | 1351 | 1358 | 0.26 |
| geranyl acetate | | 1386 | 1387 | 0.21 |
| α-humulene | | 1455 | 1460 | 0.2 |
| α-terpineol | | 1190 | 1191 | 0.19 |
| *p*-cymene | | 1024 | 1028 | 0.17 |
| *cis*-linalool oxide | | 1072 | 1072 | 0.14 |
| myrcene | | 989 | 991 | 0.12 |
| γ-muurolene | | 1477 | 1482 | 0.11 |
| *cis*-β-ocimene | | 1038 | 1038 | 0.05 |
| α-gurjunene | | 1409 | 1412 | 0.04 |
| β-pinene | | 975 | 976 | 0.03 |
| limonene | | 1029 | 1029 | 0.03 |
|  | **TOTAL** | | | **92.16** |

Experimental retention indices and literature retention indices (HP-5 column) according to NIST 14 (National Institute of Standards and Technology, USA; 14^th^ edition) library database [1], Babushok et al. [2].

**Table S3.** Composition of the essential oil of *Lavandula angustifolia*.

| **Compound** | **Lit. LRI** | **Exp. LRI** | **Area %** |
| --- | --- | --- | --- |
| linalool | 1099 | 1103 | 39.69 |
| linalyl acetate | 1263 | 1266 | 26.39 |
| *cis*-β-ocimene | 1038 | 1041 | 8.51 |
| terpinen-4-ol | 1177 | 1181 | 3.95 |
| lavandulyl acetate | 1290 | 1295 | 2.43 |
| β-caryophyllene | 1420 | 1426 | 2.1 |
| α-terpineol | 1190 | 1193 | 1.5 |
| α-humulene | 1455 | 1461 | 1.36 |
| *trans*-β-ocimene | 1051 | 1049 | 1.22 |
| α-fenchol | 1112 | 1117 | 1.13 |
| myrcene | 989 | 992 | 1.06 |
| geranyl acetate | 1386 | 1387 | 0.99 |
| borneol | 1066 | 1168 | 0.86 |
| limonene | 1029 | 1029 | 0.82 |
| lavandulol | 1170 | 1172 | 0.76 |
| 1,8-cineole | 1032 | 1031 | 0.65 |
| 3-octanone | 984 | 987 | 0.6 |
| neryl acetate | 1368 | 1369 | 0.52 |
| caryophyllene oxide | 1589 | 1593 | 0.29 |
| *trans*-linalool oxide | 1091 | 1088 | 0.21 |
| α-pinene | 936 | 933 | 0.2 |
| camphor | 1143 | 1145 | 0.2 |
| *p*-cymene | 1024 | 1024 | 0.17 |
| γ-terpinene | 1060 | 1059 | 0.17 |
| α-thujene | 928 | 927 | 0.13 |
| β-pinene | 975 | 976 | 0.11 |
| *cis*-linalool oxide | 1072 | 1073 | 0.09 |
| *p*-cymen-8-ol | 1186 | 1185 | 0.09 |
| α-phellandrene | 1004 | 1005 | 0.07 |
| camphene | 950 | 947 | 0.05 |
| sabinene | 973 | 973 | 0.05 |
| γ-cadinene | 1523 | 1521 | 0.05 |
| **TOTAL** | | | **96.76** |

Experimental retention indices and literature retention indices (HP-5 column) according to NIST 14 (National Institute of Standards and Technology, USA; 14^th^ edition) library database [1], Babushok et al. [2].

**Table S4.** Composition of the essential oil of *Lavandula hybrida.*

| **Compound** | **Lit. LRI** | **Exp. LRI** | **Area %** |
| --- | --- | --- | --- |
| linalool | 1099 | 1103 | 35.16 |
| linalyl acetate | 1263 | 1266 | 27.97 |
| camphor | 1143 | 1147 | 6.82 |
| 1,8-cineole | 1032 | 1032 | 4.74 |
| terpinen-4-ol | 1177 | 1181 | 3.48 |
| borneol | 1066 | 1169 | 3.03 |
| lavandulyl acetate | 1290 | 1295 | 2.22 |
| β-caryophyllene | 1420 | 1426 | 1.72 |
| α-humulene | 1455 | 1462 | 1.24 |
| myrcene | 989 | 992 | 0.93 |
| lavandulol | 1170 | 1171 | 0.87 |
| α-terpineol | 1190 | 1193 | 0.87 |
| *cis*-β-ocimene | 1038 | 1039 | 0.83 |
| *p*-cymene | 1024 | 1029 | 0.65 |
| germacrene D | 1481 | 1486 | 0.55 |
| geranyl acetate | 1386 | 1387 | 0.54 |
| *trans*-β-ocimene | 1051 | 1049 | 0.5 |
| α-pinene | 936 | 933 | 0.4 |
| β-pinene | 975 | 976 | 0.39 |
| myrtenal | 1196 | 1195 | 0.39 |
| *trans*-linalool oxide | 1091 | 1089 | 0.34 |
| camphene | 950 | 947 | 0.28 |
| neryl acetate | 1368 | 1369 | 0.28 |
| α-fenchol | 1112 | 1116 | 0.26 |
| octen-3-ol | 981 | 980 | 0.23 |
| γ-cadinene | 1523 | 1522 | 0.22 |
| *cis*-linalool oxide | 1072 | 1073 | 0.2 |
| *trans*-α-bergamotene | 1444 | 1441 | 0.19 |
| δ-cadinene | 1524 | 1530 | 0.16 |
| α-terpinene | 1017 | 1015 | 0.14 |
| sabinene | 973 | 973 | 0.12 |
| caryophyllene oxide | 1589 | 1593 | 0.12 |
| γ-terpinene | 1060 | 1059 | 0.11 |
| *trans*-sabinene hydrate | 1064 | 1067 | 0.11 |
| α-thujene | 928 | 927 | 0.08 |
| δ-3-carene | 1012 | 1010 | 0.07 |
| α-phellandrene | 1004 | 1005 | 0.04 |
| α-gurjunene | 1409 | 1411 | 0.03 |
| **TOTAL** | | | **96.31** |

Experimental retention indices and literature retention indices (HP-5 column) according to NIST 14 (National Institute of Standards and Technology, USA; 14^th^ edition) library database [1], Babushok et al. [2].

**Table S5.** Composition of the essential oil of *Citrus limon.*

| **Compound** | **Lit. LRI** | **Exp. LRI** | **Area %** |
| --- | --- | --- | --- |
| limonene | 1029 | 1035 | 67.8 |
| β-pinene | 975 | 978 | 14.82 |
| γ-terpinene | 1060 | 1061 | 8.61 |
| α-pinene | 936 | 933 | 2.09 |
| myrcene | 989 | 992 | 1.53 |
| geranial | 1278 | 1275 | 1.19 |
| pulegone | 1244 | 1246 | 0.8 |
| α-thujene | 928 | 926 | 0.36 |
| terpinolene | 1090 | 1088 | 0.35 |
| neryl acetate | 1368 | 1368 | 0.27 |
| α-terpinene | 1017 | 1017 | 0.25 |
| *p*-cymene | 1024 | 1026 | 0.18 |
| α-terpineol | 1190 | 1191 | 0.14 |
| geranyl acetate | 1386 | 1386 | 0.14 |
| β-caryophyllene | 1420 | 1425 | 0.14 |
| *trans*-β-ocimene | 1051 | 1049 | 0.13 |
| α-phellandrene | 1004 | 1005 | 0.08 |
| linalool | 1099 | 1101 | 0.08 |
| terpinen-4-ol | 1177 | 1178 | 0.08 |
| camphene | 950 | 947 | 0.07 |
| citronellol | 1237 | 1233 | 0.04 |
| menthone | 1154 | 1155 | 0.03 |
| α-humulene | 1455 | 1460 | 0.03 |
| α-selinene | 1495 | 1503 | 0.03 |
| α-muurolene | 1499 | 1506 | 0.03 |
| **TOTAL** | | | **99.27** |

Experimental retention indices and literature retention indices (HP-5 column) according to NIST 14 (National Institute of Standards and Technology, USA; 14^th^ edition) library database [1], Babushok et al. [2].

**Table S6.** Composition of the essential oil of *Mentha piperita.*

| **Compound** | **Lit. LRI** | **Exp. LRI** | **Area %** |
| --- | --- | --- | --- |
| menthol | 1183 | 1183 | 36 |
| menthone | 1154 | 1160 | 28.07 |
| neomenthol | 1166 | 1169 | 9.56 |
| 1,8-cineole | 1032 | 1032 | 6.47 |
| menthyl acetate | 1295 | 1298 | 4.8 |
| β-caryophyllene | 1420 | 1426 | 2.95 |
| limonene | 1029 | 1029 | 2.57 |
| β-pinene | 975 | 976 | 1.42 |
| α-pinene | 936 | 933 | 0.9 |
| pulegone | 1244 | 1244 | 0.9 |
| piperitone | 1254 | 1259 | 0.65 |
| α-terpineol | 1190 | 1194 | 0.44 |
| sabinene | 973 | 973 | 0.37 |
| α-humulene | 1455 | 1460 | 0.32 |
| caryophyllene oxide | 1589 | 1593 | 0.28 |
| *p*-cymene | 1024 | 1024 | 0.18 |
| β-bourbonene | 1384 | 1389 | 0.18 |
| myrcene | 989 | 992 | 0.16 |
| viridiflorene | 1496 | 1504 | 0.14 |
| linalool | 1099 | 1102 | 0.13 |
| camphene | 950 | 948 | 0.12 |
| isopulegol | 1148 | 1148 | 0.12 |
| 3-octanol | 996 | 997 | 0.11 |
| δ-elemene | 1337 | 1341 | 0.1 |
| α-copaene | 1376 | 1380 | 0.09 |
| germacrene D | 1481 | 1485 | 0.08 |
| carvone | 1245 | 1249 | 0.07 |
| γ-terpinene | 1060 | 1058 | 0.05 |
| *trans*-sabinene hydrate | 1064 | 1066 | 0.05 |
| geranyl formate | 1300 | 1306 | 0.05 |
| α-terpinyl acetate | 1350 | 1354 | 0.05 |
| *trans*-linalool oxide | 1091 | 1088 | 0.04 |
| *trans*-pinocarveol | 1137 | 1138 | 0.04 |
| δ-cadinene | 1524 | 1530 | 0.04 |
| *cis*-linalool oxide | 1072 | 1073 | 0.03 |
| **TOTAL** | | | **97.53** |

Experimental retention indices and literature retention indices (HP-5 column) according to NIST 14 (National Institute of Standards and Technology, USA; 14^th^ edition) library database [1], Babushok et al. [2].

**Table S7.** Composition of the essential oil of *Melaleuca leucadendron.*

| **Compound** | **Lit. LRI** | **Exp. LRI** | **Area %** |
| --- | --- | --- | --- |
| 1,8-cineole | 1032 | 1039 | 77.3 |
| α-terpineol | 1190 | 1195 | 5.56 |
| α-pinene | 936 | 934 | 3.61 |
| terpinen-4-ol | 1177 | 1181 | 2.93 |
| linalool | 1099 | 1103 | 1.94 |
| *p-*cymene | 1024 | 1026 | 1.28 |
| γ-terpinene | 1060 | 1060 | 0.96 |
| β-caryophyllene | 1420 | 1426 | 0.47 |
| borneol | 1066 | 1166 | 0.45 |
| myrcene | 989 | 993 | 0.39 |
| β-pinene | 975 | 976 | 0.36 |
| α-terpinene | 1017 | 1018 | 0.24 |
| α-phellandrene | 1004 | 1006 | 0.22 |
| terpinolene | 1090 | 1089 | 0.15 |
| geranyl formate | 1300 | 1305 | 0.1 |
| sabinene | 973 | 973 | 0.06 |
| camphene | 950 | 948 | 0.03 |
| **TOTAL** | | | **96.05** |

Experimental retention indices and literature retention indices (HP-5 column) according to NIST 14 (National Institute of Standards and Technology, USA; 14^th^ edition) library database [1], Babushok et al. [2].

**Table S8.** Composition of the essential oil *GL mix.*

| **Compound** | **Lit. LRI** | **Exp. LRI** | **Area %** |
| --- | --- | --- | --- |
| limonene | 1029 | 1031 | 23.8 |
| carvacrol | 1301 | 1309 | 14.36 |
| 1,8-cineole | 1032 | 1034 | 9.4 |
| *p*-cymene | 1024 | 1027 | 8.14 |
| terpinen-4-ol | 1177 | 1181 | 6.02 |
| geraniol | 1259 | 1264 | 5.6 |
| γ-terpinene | 1060 | 1060 | 5.36 |
| linalool | 1099 | 1103 | 5.2 |
| menthyl acetate | 1295 | 1297 | 3.43 |
| β-pinene | 975 | 977 | 2.67 |
| α-pinene | 936 | 934 | 2.57 |
| α-terpineol | 1190 | 1193 | 2.43 |
| α-terpinene | 1017 | 1018 | 1.96 |
| myrcene | 989 | 993 | 1.12 |
| β-caryophyllene | 1420 | 1426 | 1.03 |
| terpinolene | 1090 | 1089 | 0.75 |
| *cis*-β-ocimene | 1038 | 1039 | 0.65 |
| borneol | 1066 | 1167 | 0.6 |
| sabinene | 973 | 974 | 0.43 |
| *trans*-β-ocimene | 1051 | 1049 | 0.4 |
| camphor | 1143 | 1146 | 0.35 |
| camphene | 950 | 948 | 0.29 |
| α-phellandrene | 1004 | 1005 | 0.29 |
| neral | 1245 | 1250 | 0.27 |
| geranial | 1278 | 1278 | 0.17 |
| α-thujene | 928 | 927 | 0.15 |
| α-fenchol | 1112 | 1115 | 0.15 |
| thymol | 1295 | 1299 | 0.13 |
| *cis*-linalool oxide | 1072 | 1070 | 0.12 |
| geranyl acetate | 1386 | 1387 | 0.12 |
| α-humulene | 1455 | 1461 | 0.12 |
| neryl acetate | 1368 | 1369 | 0.08 |
| *trans*-linalool oxide | 1091 | 1086 | 0.03 |
| **TOTAL** | | | **98.19** |

Experimental retention indices and literature retention indices (HP-5 column) according to NIST 14 (National Institute of Standards and Technology, USA; 14^th^ edition) library database [1], Babushok et al. [2].

**Table S9.** Composition of the essential oil of *Cymbopogon nardus.*

| **Compound** | **Lit. LRI** | **Exp. LRI** | **Area %** |
| --- | --- | --- | --- |
| citronellal | 1157 | 1161 | 21.19 |
| camphene | 950 | 949 | 13.72 |
| limonene | 1029 | 1031 | 13.11 |
| geraniol | 1259 | 1265 | 12.05 |
| citronellol | 1237 | 1239 | 8.59 |
| α-pinene | 936 | 934 | 5.38 |
| citronellyl acetate | 1352 | 1357 | 1.65 |
| terpinolene | 1090 | 1088 | 1.59 |
| linalool | 1099 | 1102 | 1.46 |
| geranyl acetate | 1386 | 1387 | 1.43 |
| α-terpinene | 1017 | 1017 | 1.37 |
| δ-cadinene | 1524 | 1531 | 1.36 |
| β-elemene | 1390 | 1396 | 1.35 |
| germacrene D | 1481 | 1485 | 1.21 |
| α-thujene | 928 | 926 | 1.13 |
| isopulegol | 1156 | 1146 | 0.96 |
| *p*-cymene | 1024 | 1025 | 0.94 |
| sabinene | 973 | 975 | 0.68 |
| myrcene | 989 | 992 | 0.49 |
| γ-cadinene | 1523 | 1521 | 0.39 |
| α-phellandrene | 1004 | 1004 | 0.37 |
| α-terpineol | 1190 | 1192 | 0.33 |
| neral | 1245 | 1247 | 0.3 |
| borneol | 1066 | 1168 | 0.19 |
| γ-terpinene | 1060 | 1058 | 0.18 |
| cubenol | 1640 | 1641 | 0.18 |
| terpinen-4-ol | 1177 | 1185 | 0.15 |
| 3-octanone | 984 | 987 | 0.14 |
| β-caryophyllene | 1420 | 1424 | 0.1 |
| α-humulene | 1455 | 1460 | 0.09 |
| α-muurolene | 1499 | 1500 | 0.08 |
| α-fenchol | 1112 | 1113 | 0.06 |
| lavandulol | 1170 | 1171 | 0.06 |
| geranial | 1278 | 1280 | 0.05 |
| α-copaene | 1376 | 1380 | 0.05 |
| β-pinene | 975 | 974 | 0.04 |
| *trans*-β-ocimene | 1051 | 1048 | 0.04 |
| neryl acetate | 1368 | 1367 | 0.04 |
| α-selinene | 1495 | 1499 | 0.04 |
| **TOTAL** | | | **98.19** |

Experimental retention indices and literature retention indices (HP-5 column) according to NIST 14 (National Institute of Standards and Technology, USA; 14^th^ edition) library database [1], Babushok et al. [2].

**Table S10.** Composition of the essential oil of *Eucaliptus globulus.*

| **Compound** | **Lit. LRI** | **Exp. LRI** | **Area %** |
| --- | --- | --- | --- |
| 1,8-cineole | 1032 | 1040 | 91.44 |
| α-pinene | 936 | 934 | 2.91 |
| γ-terpinene | 1060 | 1060 | 2.06 |
| α-phellandrene | 1004 | 1006 | 0.86 |
| myrcene | 989 | 993 | 0.68 |
| β-pinene | 975 | 976 | 0.5 |
| α-terpinene | 1017 | 1018 | 0.18 |
| terpinen-4-ol | 1177 | 1179 | 0.05 |
| *trans*-β-ocimene | 1051 | 1050 | 0.03 |
| camphene | 950 | 947 | 0.02 |
| **TOTAL** | | | **98.73** |

Experimental retention indices and literature retention indices (HP-5 column) according to NIST 14 (National Institute of Standards and Technology, USA; 14^th^ edition) library database [1], Babushok et al. [2].

**Figure S1.** Graphical overview of the effects of all test compounds on morpho-functional parameters on swine spermatozoa upon short- (3h) and long-term (120h) storage.

**
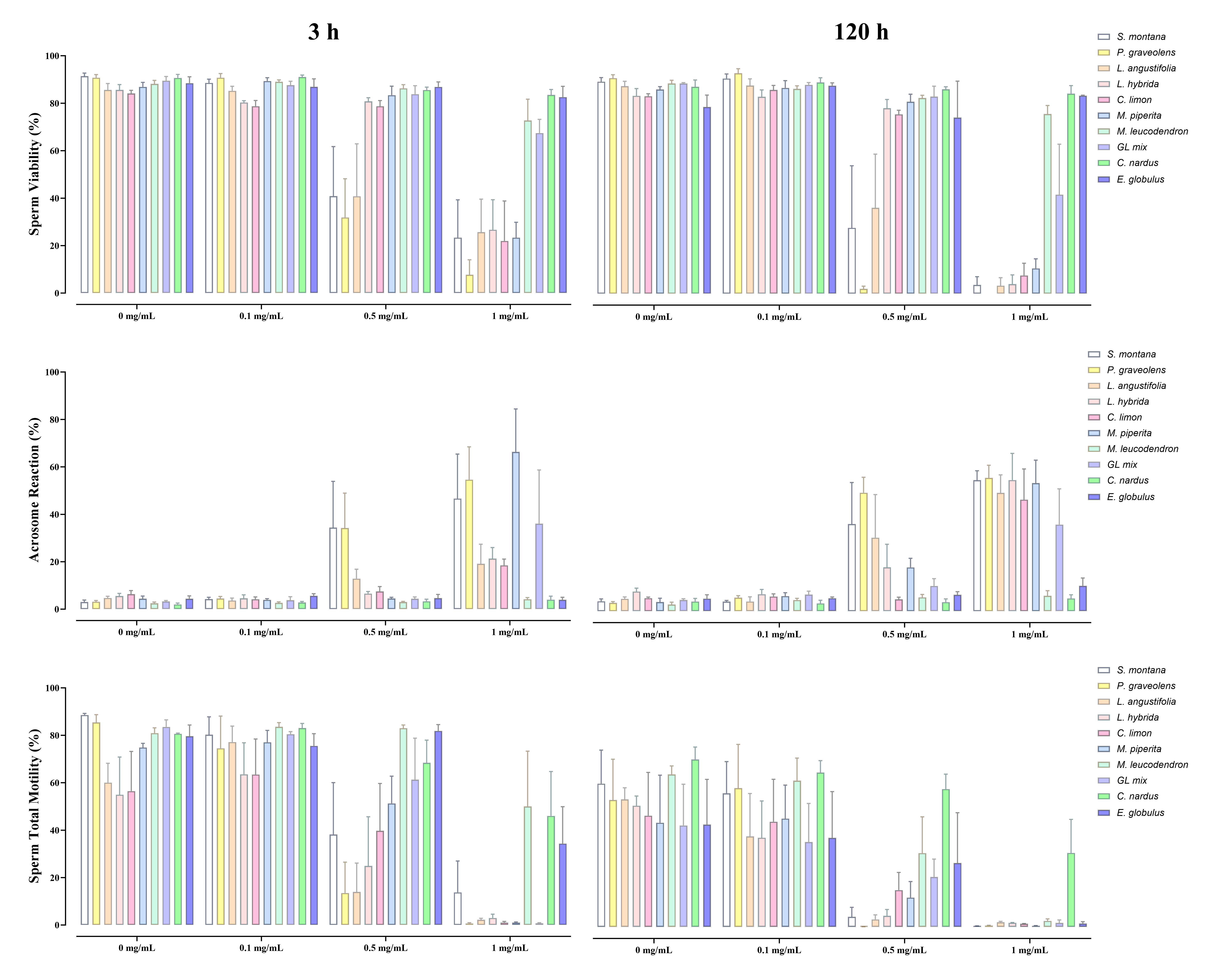
**

[1] National Institute of Standards and Technology Library Database. Available online: https://webbook.nist.gov/ (accessed on 25 October 2023).

[2] Babushok, V.I.; Zenkevich, I.G. Retention indices for most frequently reported essential oil compounds in GC. Chromatographia 2009, 69, 257–269.
